# Isradipine attenuates MPTP-induced dopamine neuron degeneration by inhibiting up-regulation of L-type calcium channels and iron accumulation in the substantia nigra of mice

**DOI:** 10.18632/oncotarget.17618

**Published:** 2017-05-04

**Authors:** Qi-Min Wang, Yu-Yu Xu, Shang Liu, Ze-Gang Ma

**Affiliations:** ^1^ Department of Physiology, School of Basic Medicine, Medical College of Qingdao University, Qingdao, China; ^2^ Institute of Brain Science and Disorders, Qingdao University, Qingdao, China

**Keywords:** L-type calcium channel, isradipine, dopamine neuron, iron accumulation, Parkinson's disease

## Abstract

The aim of this study is to investigate the effects of L-type calcium channels (LTCCs) on MPTP-induced dopamine (DA) neuron degeneration and iron accumulation in the substantia nigra (SN) of mice. By real-time PCR and western blots, we first quatified expressions of L-type Cav1.2 and Cav1.3 calcium channel α1 subunits in the SN of experimental mice treated with MPTP. We found that the expressions of Cav1.2 and Cav1.3 calcium channel α1 subunits markedly increased after MPTP treatment for 2 and 3 weeks. Secondly, we observed the effects of isradipine, a LTCC antagonist, on MPTP-induced DA neuron degeneration and iron accumulation in the SN. Our results showed that isradipine treatment prevented against MPTP-induced Cav1.2 and Cav1.3 calcium channel α1 subunits up-regulation in the SN. We also found that isradipine prevented against MPTP-induced DA neuron depletion in the SN and partly restored the DA content in the striatum. Moreover, we found that isradipine inhibited the increase of iron positive cells in the SN of the MPTP-treated mice. Finally, we investigated the effects of isradipine on cellular iron accumulation in the dopaminergic MES23.5 cell line. Our studies showed that MPP^+^ treatment accelerated iron influx in the MES23.5 cells. Treatment with Bayk8644 further aggravated iron accumulation. Treatment with isradipine prevented against MPP^+^-induced iron influx in the MES23.5 cells. These results suggest that up-regulation of LTCCs may be responsible for the DA neuron degeneration in the MPTP-treated mice, The LTCCs may directly contribute to iron influx into DA neurons, and isradipine may suppress cellular iron accumulation and prevents neurodegeneration.

## INTRODUCTION

Parkinson's disease (PD) is one of the slowly progressing neurodegenerative disorders characterized by resting tremors, bradykinesia, and rigidity. The pathogenesis of PD is selective loss of dopamine (DA) neurons in the substantia nigra pars compacta (SNpc) and exhaustion of dopamine in the striatum [1]. Many factors have been implicated in the pathological process of DA neuron degeneration, which include but is not limited to oxidative stress, neuroinflammation, apoptosis, increased iron leave, excito-toxicity, and decreased proteasome function [1–4].

Increasing evidence has indicated that iron plays a key role in the pathogenesis of DA neuron degeneration. Substantially increased content of iron in the PD brains was observed and demonstrated in previous studies. This iron increase only occured in the SN but not in the ventral tagmental area (VTA) [5–10]. The exceedingly increased iron may lead to neuronal death by reacting with oxygen and produces highly reactive hydroxyl radicals. Increased iron content may damage lipids, carbohydrates, proteins and nucleic acids, thereby inducing DA neuron degeneration in the SN [11]. However, the underlying mechanisms of iron selective accumulation in the SN remain unclear.

In addition to the iron selective accumulation in the SN, growing evidence suggests that the L-type calcium channels (LTCCs) may play an important role in the selective DA neuron degeneration in the SN [12–14]. The potential linkage of LTCCs to PD is strengthened by epidemiological studies, which showed a decreased risk for PD in humans who were treated for hypertension with dihydropyridines (DHPs) [4, 15, 16]. L-type Cav1.2 and Cav1.3 calcium channels, especially the Cav1.2 calcium channels are abundant in neurons [16]. Nevertheless, the LTCCs are responsible for mitochondrial oxidant stress; and increased vulnerability in the SN DA neurons is largely attributable to the expression of Cav1.3 calcium channels [17, 18]. DA neurons in the SN rely on sodium channels for pacemaking at their juvenile, but rely on L-type Cav1.3 calcium channels to drive autonomous pacemaking at the progress of adulthood; whereas, DA neurons in the VTA rely on sodium channels for pacemaking [19, 20]. Previous studies showed that Ca^2+^ enter DA neurons via the continuous opening of Cav1.3 calcium channels during pacemaking. This could be a reason why SN DA neurons exhibit vulnerability when stressed [3, 21]. Further support for an involvement of LTCCs in the pathogenesis of PD, comes from the finding that Cav1 subtype was elevated in the brain regions affected in PD, resulting in a generally higher level of Cav1.3 subtype expression relative to that of the Cav1.2 subtype [22]. The increased expression and therefore, presumably increased cellular use of Cav1 subtypes could lead to increased metabolic stress on neurons. Yet, to date, the dynamic variations of Cav1 subtypes expression in the pathological process of PD are still unknown.

In the last few years, LTCC was reported to provide a major pathway for iron entry into cardiomyocytes [23, 24]. LTCC may also provide an alternative route for iron import to neuronal cells [25]. Previous study in my laboratory demonstrated that LTCC blocker nifedipine attenuated iron deposit in the SN during iron-overload condition [26]. We proposed that LTCC might partly mediate iron selective accumulation in the SN. In the present study, we first quantified the expressions of L-type Cav1.2 and Cav1.3 calcium channel α1 subunits in the SN of experimental mice treated with MPTP. Secondly, we observed the effects of isradipine, a LTCC blocker, on the MPTP-induced neurotoxicity and iron accumulation in the mice.

## RESULTS

### Isradipine prevented against MPTP-induced motor coordination ability impairment assessed by rotarod test

**Figure 1:**
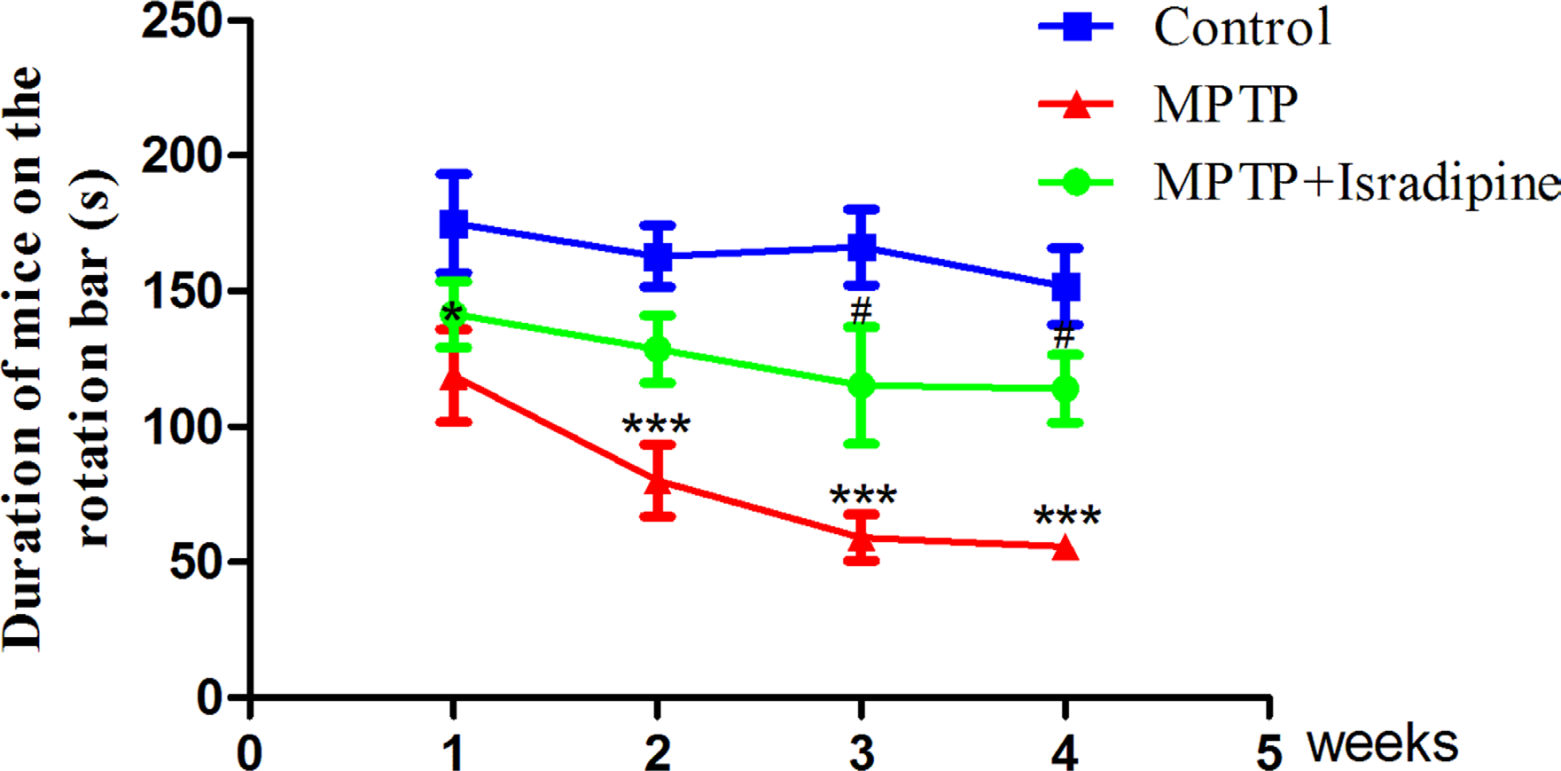
Isradipine prevented against MPTP-induced motor coordination ability impairment After treatment with MPTP for 1-4 weeks, the time on the rod of the mice significantly reduced compared with the controls. This effect was partly restored by co-treatment with isradipine (*P < 0.05, ***P < 0.001, compared with control; ^#^*P* < 0.05, compared with MPTP treatment group; *n* = 10).

As shown in Figure 1, after 1–4 weeks of MPTP treatment, the time on the rod of the MPTP treated mice reduced significantly compared with the control mice. The group, which was treated with isradipine followed by MPTP, partly resist this reduction in the 3- and 4-week treated subgroups. Time on the rod of the isradipine treated mice from the 1- and 2-week treatment subgroups also showed an increasing trend, however, no significant changes were observed (*P < 0.05, ***P < 0.001 compared with control; ^#^*P* < 0.05 compared with MPTP treatment group, *n* = 10).

### Expressions of Cav1.2 and Cav1.3 α1 subunits altered with the progression of PD

**Figure 2:**
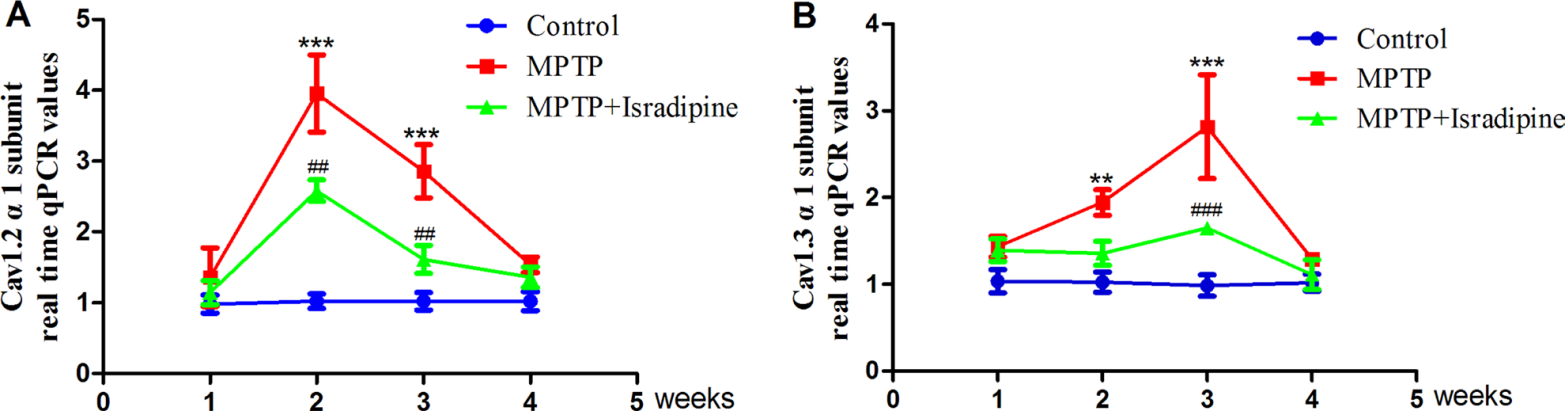
mRNA expressions of Cav1.2 and Cav1.3 α1 subunits increased in the SN of MPTP-treated mice The mRNA expressions of Cav1.2 (**A**) and Cav1.3 (**B**) α1 subunits in the SN markedly increased compared with the controls after treated with MPTP for 2 and 3 weeks. The peak of Cav1.2 α1 subunit mRNA appeared after MPTP treated for 2 weeks, while the peak of Cav1.3 α1 subunit mRNA appeared after MPTP treated for 3 weeks. This effect was partly restored by co-treatment with isradipine (**P < 0.01, ***P < 0.001, compared with control; ^##^*P* < 0.01, ^###^*P* < 0.001, compared with MPTP treatment group; *n* = 5).

After 2 and 3 weeks of MPTP treatment, the mRNA expressions of Cav1.2 and Cav1.3 α1 subunits in the SN markedly increased compared with the controls. However, we cannot observe significant changes after MPTP treatment for 1 or 4 weeks compared with the controls. As for the Cav1.2 α1 subunit, the peak of its mRNA expression appeared after MPTP treated for 2 weeks (Figure 2A). While the peak of Cav1.3 α1 subunit mRNA expression appeared after MPTP treatment for 3 weeks (Figure 2B). Treatment with isradipine conferred significant protection against MPTP-induced up-regulation (**P < 0.01, ***P < 0.001, compared with control; ^##^*P* < 0.01, ^###^*P* < 0.001, compared with MPTP treatment group; *n* = 5).

**Figure 3:**
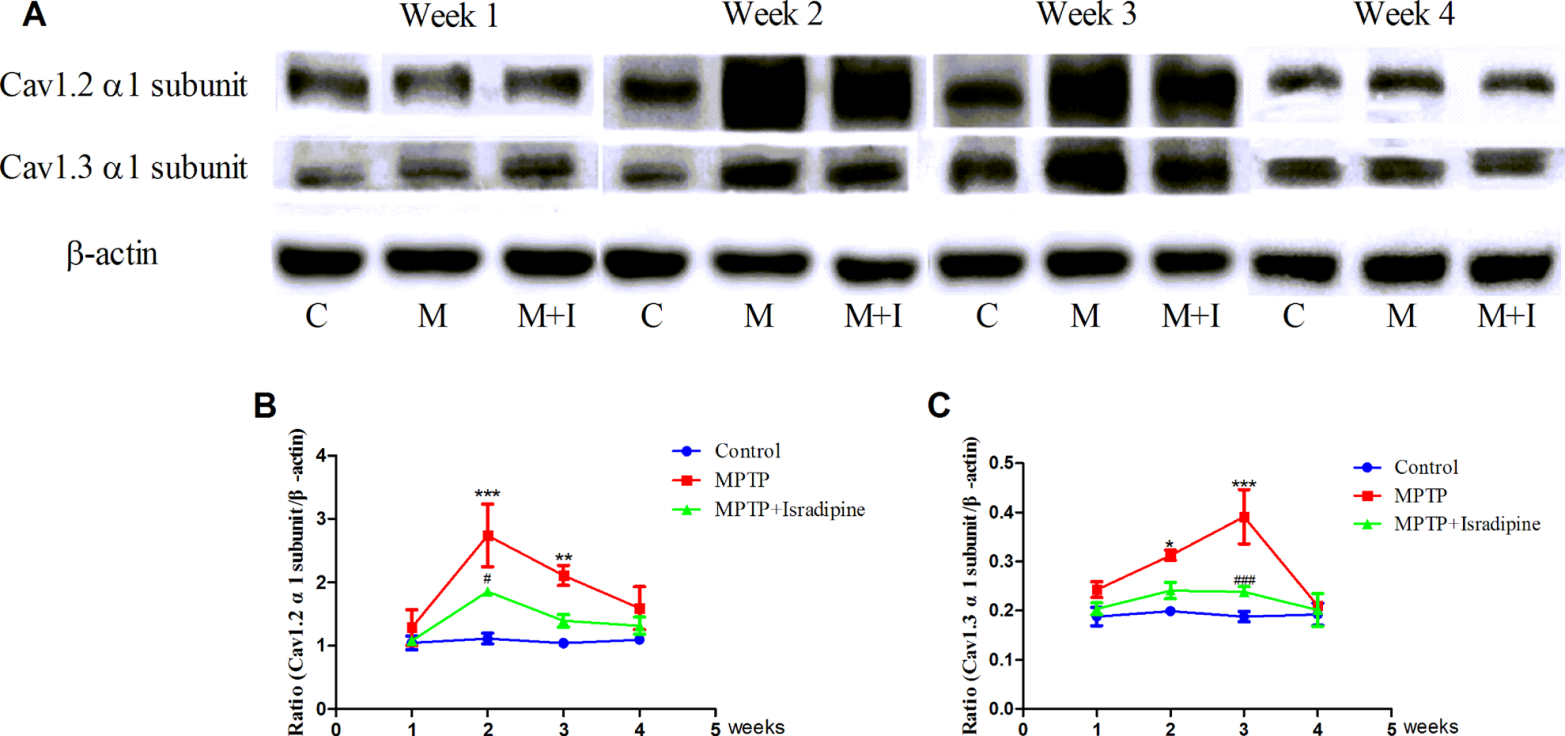
Protein expressions of Cav1.2 and Cav1.3 α1 subunits increased in the SN of MPTP-treated mice (**A**) Original bands showed the Cav1.2, Cav1.3 α1 subunits and β-actin expressions in different groups (C, Control group; M, MPTP treatment group; M+I, MPTP and isradipine co-treatment group). (**B**–**C**) The same tendency of protein expression with their mRNA in the SN were observed. No significant changes at the 1 and 4 weeks, while a significant up-regulation at the 2 and 3 weeks were observed in the MPTP treatment group compared with control. These results could be partly restored by isradipine (*P < 0.05, **P < 0.01, ***P < 0.001, compared with control; ^#^*P* < 0.05, ^###^*P* < 0.001, compared with MPTP treatment group; *n* = 5).

We also investigated the expressions of Cav1.2 and Cav1.3 α1 subunits by western blots. As shown in Figure 3, the same tendency of protein expressions with their mRNA in the SN were observed. No significant changes at 1 and 4 weeks, while a significant up-regulation was observed at 2 and 3 weeks in the MPTP treated group compared with the controls. These results could be partly restored by isradipine (*P < 0.05, **P < 0.01, ***P < 0.001, compared with control; ^#^*P* < 0.05, ^###^*P* < 0.001, compared with MPTP treatment group; *n* = 5).

### Isradipine protected against MPTP-induced decrease of TH positive neurons in the SN of mice

**Figure 4:**
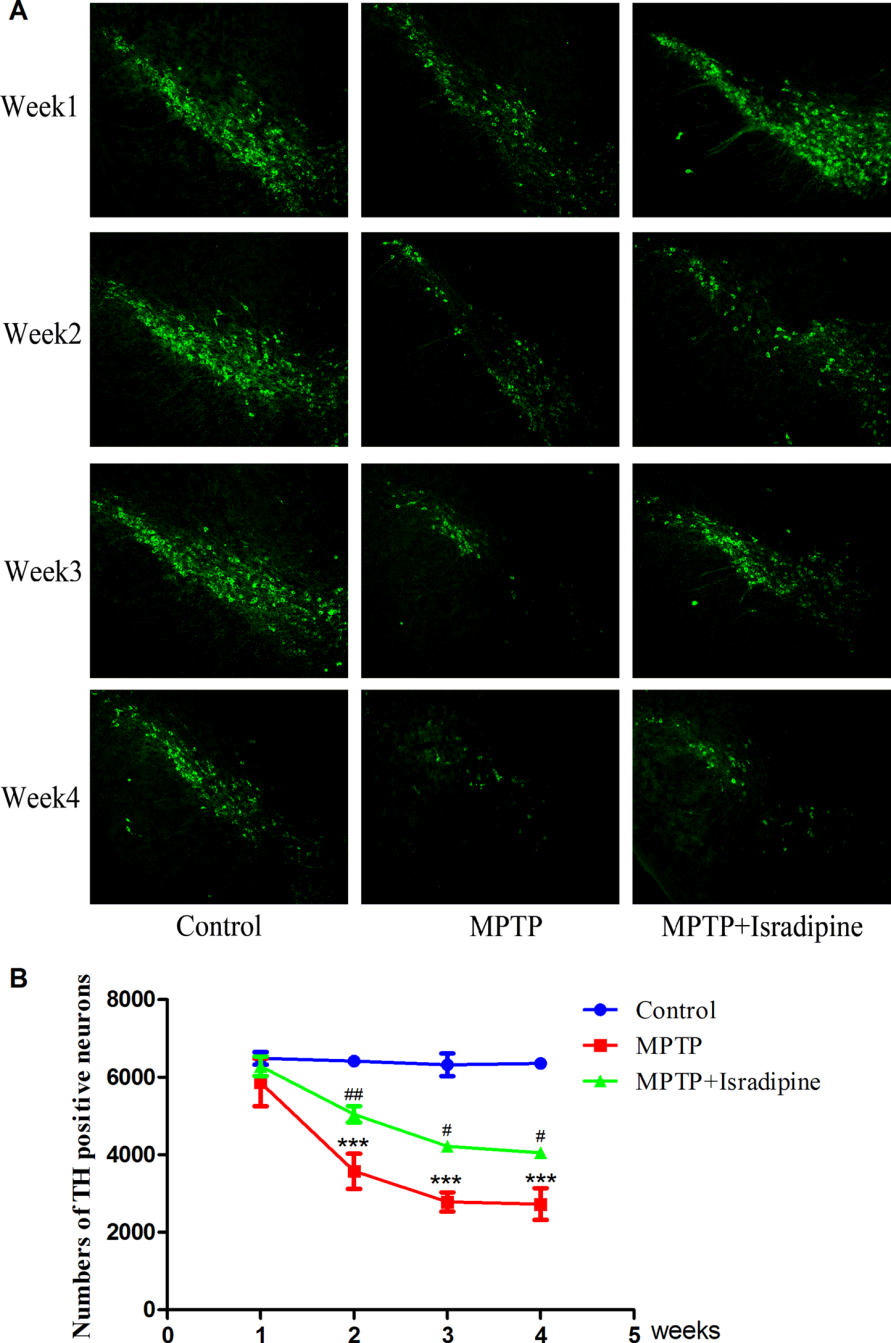
Isradipine inhibited the decrease in the numbers of TH positive neurons in the SN induced by MPTP Original figures showed the TH positive neurons in different treatment groups (**A**) Summarized data showed the numbers of TH positive neurons in different treatment groups (**B**) (*P < 0.05, ***P < 0.001, compared with control; ^#^*P* < 0.05, ^###^*P* < 0.001, compared with MPTP treatment group; *n* = 5).

The numbers of TH positive neurons were evaluated by immunohistochemistry. Figure 4A showed the fluorescence pictures of the whole SN. The summarized data of TH positive neurons were shown in Figure 4B. Compared with the controls, the TH positive neurons exhibited a progressive loss in the SN in the MPTP treated group. This effect was partly restored in the isradipine treatment group (*P < 0.05, ***P < 0.001, compared with control; ^#^*P* < 0.05, ^###^*P* < 0.001, compared with MPTP treatment group; *n* = 5).

### Isradipine protected against MPTP-induced decrease of DA content in the striatum of mice

**Figure 5:**
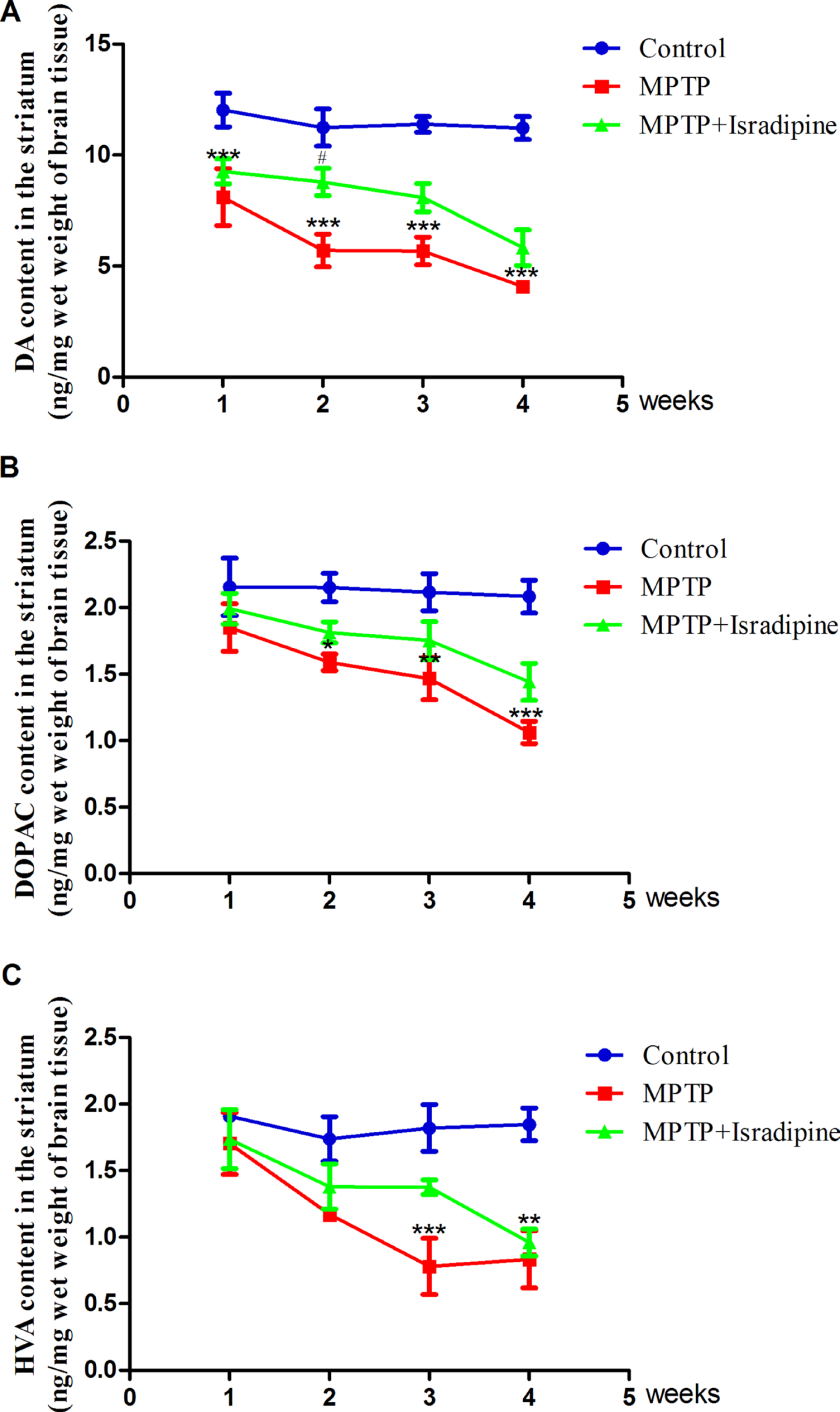
Effects of isradipine on the DA and its metabolites contents in the striatum of MPTP-treated mice The DA and its metaboites contents in the MPTP treatment group decreased compared with that of control. The dopamine content was partly restored by isradipine compared with that of MPTP treatment group. The DOPAC and HVA contents were also partly restored by isradipine, however, no significance were observed (*P < 0.05, **P < 0.01, ***P < 0.001, compared with control; ^#^*P* < 0.05, compared with MPTP treatment group; *n* = 5).

As shown in Figure 5, the mean contents of DA and its metabolites DOPAC and HVA in the striatum were significantly decreased in the MPTP treatment group compared with the controls. Co-treatment with isradipine partly restored the DA content after MPTP treated for 1 and 2 weeks. Although we found a tendency of increase in the DA content after isradipine treated for 3 and 4 weeks, no significance was observed. The DOPAC and HVA contents were also partly restored by isradipine, however, no significance was observed (*P < 0.05, **P < 0.01, ***P < 0.001, compared with control; ^#^*P* < 0.05, compared with MPTP treatment group; *n* = 5).

### Isradipine inhibited MPTP-induced increase of iron-staining cells in the SN of mice

**Figure 6:**
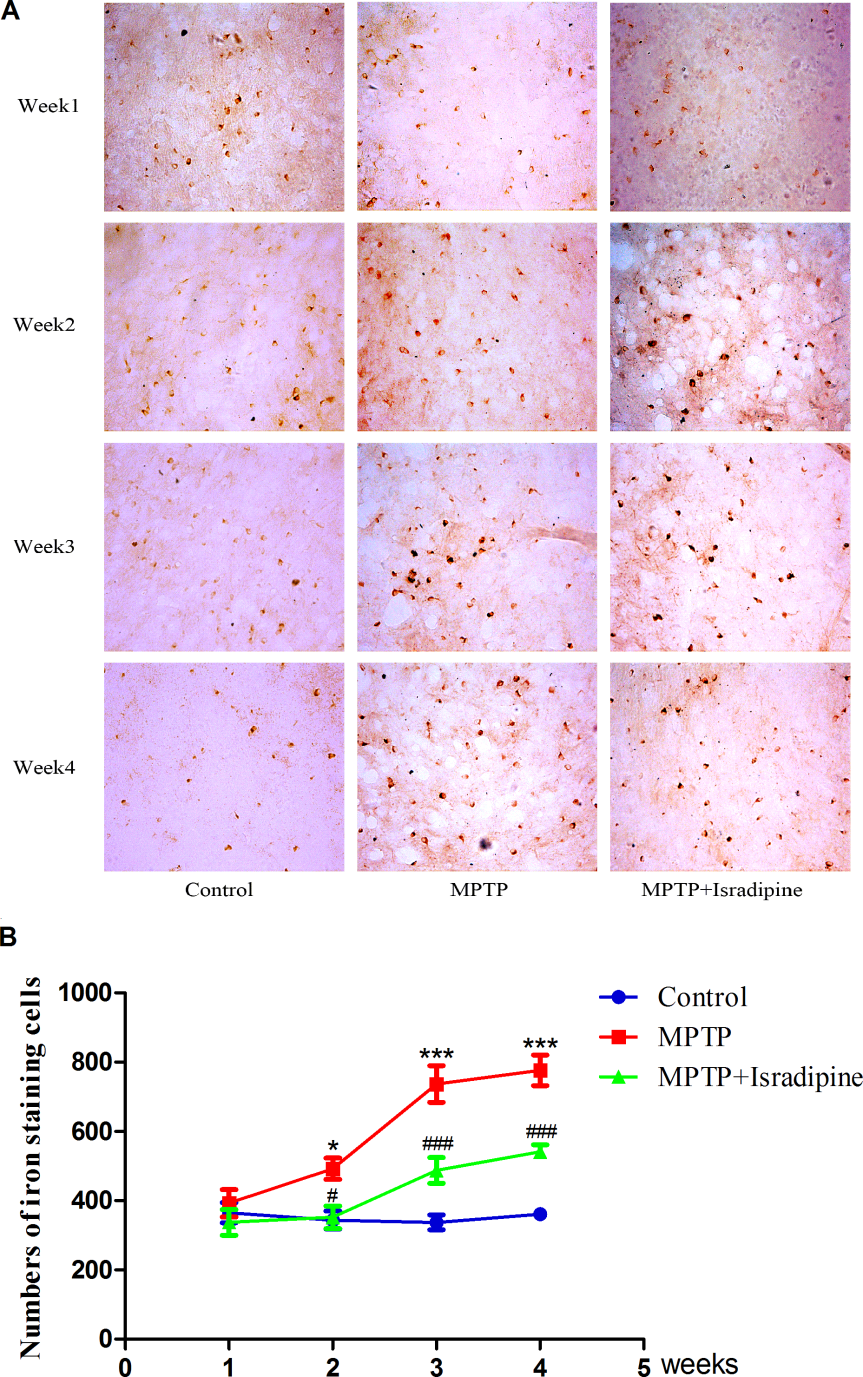
Isradipine inhibited the increase in the numbers of iron staining cells in the SN induced by MPTP Original figures showed the iron staining cells in different treatment groups (**A**) Summarized data showed the numbers of iron staining cells in different treatment groups (**B**) (*P,<0.05, ***P < 0.001, compared with control; ^#^*P* < 0.05, ^###^*P* < 0.001, compared with MPTP treatment group; *n* = 5).

The iron staining cells in the SN were detected by Perls’ iron staining. As shown in Figure 6, No changes in the numbers of iron staining cells were observed in the 4 subgroups of control group. However, a marked and time-dependent increase in the numbers of iron-staining cells were detected after MPTP treated for 2 to 4 weeks. A significant decrease of the numbers in iron-staining cells were observed in isradipine co-treatment group compared with that of MPTP-treatment group (*P < 0.05, ***P < 0.001, compared with control; ^#^*P* < 0.05, ^###^*P* < 0.001, compared with MPTP treatment group; *n* = 5).

### Isradipine inhibited MPTP-induced iron influx in the MES23.5 cells

**Figure 7:**
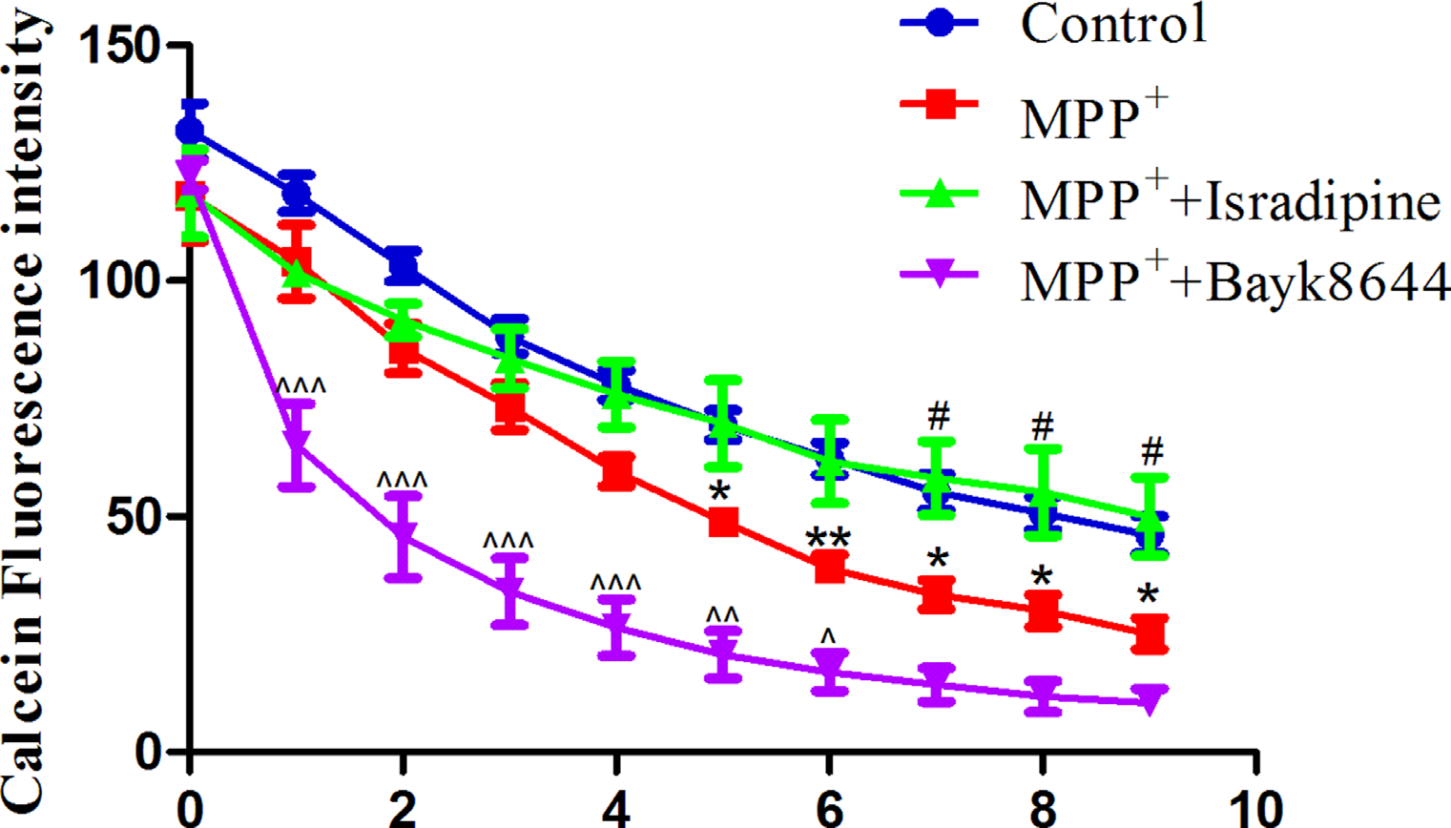
Isradipine inhibited MPP+-induced iron influx in the MES23.5 cells There was a time-dependent intracellular fluorescence quenching in control group. A rapid fluorescence quenching were detected in the MPP^+^ treatment group compared with control. The fluorescence quenching further accelerated when cells were treated with Bayk8644 compared with MPP^+^ treatment group. Fluorescence quenching in the MPP^+^ treatment group was blocked by isradipine (*P < 0.05,**P < 0.01, compared with control; ^#^*P* < 0.05, compared with MPP^+^ treatment; ^^^*P* < 0.05, ^^^^*P* < 0.01, ^^^^^*P* < 0.001, compared with MPP^+^ treatment; *n* = 6).

The intracellular iron contents in MES23.5 cells were measured by fluorescence dye calcein. Fluorescence quenching indicates that extracellular iron was transported into cells. As shown in Figure 7, there was a time-dependent intracellular fluorescence quenching with 1 mmol/L ferrous iron perfusion (control), indicating the increased intracellular iron level. When cells were treated with MPP^+^ for 24 h, a rapid fluorescence quenching were detected (MPP^+^ treatment group). The fluorescence quenching further accelerated when cells were perfused with 0.01 mmol/L Bayk8644 compared with MPP^+^ treatment group, indicating an further iron influx in these cells. Fluorescence quenching in the MPP^+^ treatment group was blocked by 0.02 mmol/L isradipine perfusion (*P < 0.05, **P < 0.01, compared with control; ^#^*P* < 0.05, compared with MPP^+^ treatment; ^^^*P* < 0.05, ^^^^*P* < 0.01, ^^^^^*P* < 0.001, compared with MPP^+^ treatment; *n* = 6).

## DISCUSSION

In the present study, we found out that isradipine, the LTCC antagonist, was able to improve motor coordinate deficit, to protect against DA neuron degeneration in the SN, and to resist against the decrease of DA content in the striatum induced by MPTP in the mice. These findings confirm the critical role of LTCCs played in the pathogenesis of DA neuron degeneration. Our results for the first time clearly showed the up-regulation of Cav1.2 and Cav1.3 α1 subunits in the SN of MPTP-induced PD mice. It has been shown that L-type Cav1.2 calcium channels were widely expressed in mouse brain (about 76.5% of Cav1.2 in total L-type α1 subunit mRNA) [27], meanwhile, a relatively lower expression of Cav1.3 calcium channels (23%) and an even lower expression of Cav1.1 and Cav1.4 calcium channels (0.5% together) were observed [27]. The DA neurons in the SN are inhabited by both Cav1.2 and Cav1.3 calcium channels α1 subunit isoforms. Recently, the Cav1.3 calcium channel's functions were identified, including pacemaking and making SN DA neurons more vulnerable to toxins [21]. Research suggested that Ca1.3 calcium channels were the cause of Ca^2+^ overload in SN DA neurons [19]. There is also evidence shows that, disregulated calcium homeostasis is one of the major factors that implicated the pathogenesis of PD [4, 7, 22, 28]. In our acquired results, Cav1.2 and Cav1.3 calcium channels increased with the progression of PD. Both type of channels are up-regulated in the SN. Therefore, with the increase of these two channels, Ca^2+^ concentration also increases in the cytoplasm, this proportional alteration results in a series of reactions accompanied by the Ca^2+^ overload. It was worth noting that the up-regulation of both channels decreased after 4-week MPTP treatment, and the expressions of both channels even returned to the control level. This observation might be attributed to the loss of many DA neurons.

More importantly, our study demonstrated the iron continuous accumulation in SN was accompanied by the progression of PD, yet isradipine reversed this phenomenon effectively in MPTP-treated mice. In MPP^+^-induced PD cell model, we also observed MPP^+^ treatment was able to accelerate iron transport in MES23.5 cells. Activation of LTCCs by Bayk8644 further accelerated this process. The isradipine that successfully inhibited iron influx in the cells, may indicate that iron might be transported across cell membrane via the LTCCs. All these results directed the fact that LTCCs participate in iron accumulation in the SN. It is known that iron plays a key role in the development of PD [29]. Prevention of iron in the SN provides an effective method to slow down or terminate the progression of PD [25]. However, the cause of the selectivity of the iron deposit in the SN remain unknown. LTCCs were reported to serve as a major pathway of iron entry into cardiomyocytes [22, 23]. Iron may enter into neurons via LTCCs as well [30]. These findings suggest that LTCCs may provide an alternative route for iron import into neuronal cells. Compared with previous studies, our study first demonstrated the persistent accumulation of iron in the SN progresses with PD progression, rather than suddenly appears at the final stage of PD. Isradipine apparently inhibits the increase of iron level in the SN and inhibits iron influx into cells. These results indicate that LTCCs at least partly contribute to the iron accumulation in the SN. However, we still do not know, whether Cav1.2 or Cav1.3 calcium channels mediated iron deposit. To date, we cannot find a DHPs which selectively blocks Cav1.2 or Cav1.3 calcium channels. Most of the DHPs, including nimodipine and nifedipine, are more effective blockers for Cav1.2 than for Cav1.3 calcium channels. However, low potency DHPs such as nimodipine could fail to antagonize Cav1.3 against MPTP on dopaminergic terminals, while Cav1.3 is still involved in terminal degeneration [15, 31]. Isradipine, one of the DHPs, from earlier studies on these drugs, has a roughly 40 fold higher affinity for Cav1.3 calcium channel than other DHPs, and has neuroprotective effect on both MPTP and 6-OHDA-induced PD models [11, 32]. We cannot rule out the fact that Cav1.3 calcium channels mediate iron deposit, which could be responsible for PD pathogenesis.

In conclusion, our study indicates that the L-type calcium channels are associated with the development and progression of DA neuron degeneration via accelerating calcium and iron accumulation. Isradipine, a calcium channel blocker, may slow down DA neuron degeneration by the blockage of Cav1.2 and/or Cav1.3 calcium channels.

## MATERIALS AND METHODS

### Materials and animal preparation

All of the reagents were purchased from Sigma Chemical Co. (St. Louis, MO, USA) unless otherwise indicated. All procedures were carried out in accordance with the National Institutes of Health Guide for the Care and Use of Laboratory Animals, and were approved by the Animal Ethics Committee of Qingdao University. For the *in vivo* experiments, 120 male C57BL/6 mice (8–10 weeks old) weighting 22–25g were included in this study. The mice were obtained from Beijing Vitalriver Laboratory Animal Technology Co. Ltd. (Beijing, China). The mice were housed for 1 week with a 12-h light-dark cycled and free access to food and water before experiments. The room temperature was adjusted to 19 ± 2°C, humidity was 60 ± 5%.

MPTP and isradipine were dissolved respectively in 0.9% saline and 2% dimethysulfoxide (DMSO). These solutions were formulated freshly before used. The mice were randomly divided into 3 groups: control group, MPTP treatment group, and isradipine with MPTP co-treatment group. Each group was divided into 4 subgroups (1, 2, 3 and 4 weeks treatment subgroups). Each subgroup has 10 mice. (1) MPTP treatment group: mice were received intraperitoneal injection of MPTP (30 mg/kg) twice per week. (2) Isradipine and MPTP co-treatment group: mice undergoing same procedures mentioned above plus isradipine with a dosage of 3 mg/kg was subcutaneous injected to mice once per day [15]. (3) Control group: the same procedures were followed except MPTP and isradipine were both replaced by 0.9% saline. In each subgroup, the mice were received rotarod behavior test after treated for 1, 2, 3 or 4 weeks respectively and then were decapitated on the next day. The brains were isolated for different assays. In each subgroup, 5 of the brains were used for TH immunofluorescent and iron staining, the others were used for real time PCR, western blots, and HPLC detection.

### Rotarod behavioral test

The rotarod behavior test was used to evaluate the ability of balance and motor coordination of mice. The diameter of the rod is 5 cm. First, the mice were placed on this rod to adapted for 2 min, then the rotarod uniform accelerated from 4–40 rpm in 5 min. During this period, if any mice drop down, the system would automatically stop and record the time that mice stayed on the rotation bar. This experiment was repeated for 3 times. The interval between each two tests was not less than 1 h.

### RNA isolation and analysis of Cav1.2 and Cav1.3 α1 subunit mRNA

To measure mRNA expression of Cav1.2 and Cav1.3 α1 subunits in the SN, mice were decapitated and the brains were isolated for further experiments. Left side of SN was isolated and put into RNase free centrifuge tubes (1.5 ml), stored at −80°C before used. Each sample was homogenized in 500 μl TRIzol reagent to purification total RNA then quantified its concentration. The total mRNA was reverse transcribed to cDNA used the first-strand cDNA synthesis kit (Thermo, Waltham, USA). The mRNA expressions were analyzed by Eppendorf system (Eppendorf, Hamburg, GER) using SYBR Green PCR Master Mix (Qiagen, GER) with 2-step PCR program (95°C for 5 s, 60°C for 10 s, 40 cycles). Each sample was performed in triplicate and values were averaged. Cycle threshold (Ct) values for target genes were normalized to the housekeeping gene β-actin. The 2^−ΔΔCt^ method was used to calculate the amount of target gene. PCRs were performed by using the following primers:

Cav1.2: Forward 5′GGTTTCGTCATTGTCAC CTTCCA3′

Reverse 5′CACTTTGTACTGGTGCTGGTTC3′

Cav1.3: Forward 5′CGCTGTTCACAGTCTCAA CTTTT3’

Reverse 5′AGGCAACGATGATGATGTAGATG3′

β-actin: Forward 5′ TGCTGTCCCTGTATGCC TCT3′

Reverse 5′TTGATGTCACGCACGATTTC3′

### Western blot

The right side of SN was digested by RIPA lysis buffer (50 mmol/L Tris-HCl; 150 mmol/L NaCl; 1% Nonidet; 0.5% deoxycholate; 1 mmol/L EDTA; and 1 mmol/L PMSF) with protease inhibitors (1 g/ml each of pepstatin, aprotinin and leupeptin) for 30 min. The protein concentration was determined by a BCA bicinchoninic acid kit. 30 μg of each protein sample was separated using 8% SDS-PAGE and transferred to PVDF membranes with a diameter of 0.45 μm. The PVDF membranes were blocked with 5% non-fat milk at 4°C overnight. The membranes were incubated with primary antibodies (ab58552, ab85491, ab5694; Abcam, Cambridge, UK), rabbit anti-mouse Cav1.2 antibody (1:200), mouse anti-mouse Cav1.3 antibody (1:500), rabbit anti-mouse β-actin antibody (1:15000) overnight at 4°C. After washed, the membranes were incubated with goat-anti rabbit and goat-anti mouse secondary antibodies conjugated with horseradish peroxidase (1:10000) at room temperature for 1 h. The antigen-antibody complexes were detected with enhanced chemiluminescence (ECL) reagent and visualized by Imager (UVP Biospectrum 810, USA).

### Immunofluorescent labeling for tyrosine hydroxylase (TH) positive neurons

After anesthetized by 8% chloral hydrate (1.6 mg/kg, i.p.), the mice were perfused with 0.9% saline for 15 min then followed by 4% paraformaldehyde (PFA) in 0.1 mol/L phosphate buffered saline (PBS, pH7.4) for 20 min. The brains were isolated and post-fixed overnight in 4% PFA and followed by cryoprotection in 30% sucrose at 4 °C for at least 72 h. After the brains sank to the bottom of the centrifuge tube, they were taken out for the next steps. 20 μm serial coronal slices containing SNpc were collected in a −20°C freezing cryostat (Leica, GER). We collected every 4th serial section as 4 sets of sections and alternate set of sections were stained for TH or iron. Each set of sections contained about 15 such sections. For TH immunohistochemical detection, all the sections were kept free-floating in 0.01 mol/L PBS (pH7.4). The brain sections were blocked with 10% normal goat serum containing 0.3% Triton-X 100 for 1 h at 37°C. Rabbit anti-mouse TH primary antibody (1:2000) was used to incubate the sections overnight at 4°C temperature (AB152; Milipore, Billerica, Massachusetts, USA). The sections were treated with Alexa Fluor^®^ 488 donkey anti-rabbit IgG (A21206; Molecular Probs, Eugene, Oregon, USA) at room temperature for 2 h. After wash, sections were pasted on glass slides. The results were analyzed by counting the numbers of TH positive neurons on a Zeiss microscope (Carl Zeiss AG, Germany). All the micrographs were quantified using Image J software, which is developed by NIH.

### HPLC-ECD to detected the contents of dopamine (DA) and its metabolites (DOPAC and HVA)

Both sides of striatum were isolated and transferred into liquid nitrogen for storage. Samples were prepared by using previously described techniques in our laboratory [33]. Briefly, the striatums were homogenized in 0.1 ml A solution (0.4 mol/L perchloric acid) and followed with centrifuge at 12000 rpm for 20 min at 4°C, then 40 μl solution B (20 mmol/L citromalic acid-potassium, 300 mmol/L dipotassium phosphate, and 2 mmol/L EDTA-2Na) were added to the supernatant. The HPLC (Waters Corp., Milford, MA, USA) was used to determine the contents of DA, DOPAC and HVA. Separation was achieved by a PE C18 reverse-phase column.

### Perls’ iron staining

Perls’ iron staining was carried out according to previous report in our laboratory [34]. Freshly prepared solution (2% HCl and 2% potassium ferrocyanide) was used to incubated the sections for 30 min. Negative control was prepared without adding the freshly prepared solution. After washing 3 times with 0.01 mol/L PBS, sections were immersed in 99% methanol containing 1% hydrogen peroxide for 20 min to quench endogenous peroxidase activity. After washed 3 times with 0.01 mol/L PBS, the slices were incubated in a solution of diaminobenzidine (DAB). All incubations and washes were performed in polypropylene troughs that had been washed in 10% HCl (< 0.02 ppm Fe) overnight and rinsed in Milli-Q water. Staining was analyzed by counting the number of positive cells at 400 × magnification on an Olympus microscope. We used the average number of positive cells in four non-overlapped fields per slide to conduct iron density. All counts were carried out blindly by a person unaware of the groups of the animals.

### Cell culture

The MES23.5 cells were a generous gift from Dr. WD Le (Baylor College of Medicine, TX, USA). It is a dopaminergic cell line hybridized from murine neuroblastoma-glioma N18TG2 cells with rat mesencephalic neurons, which exhibits several properties that are similar to the primary neurons originated in the SN [35]. Cells were cultured in DMEM/F12 growth medium supplemented with 10% FBS, 2% Sato’s, 100 units/ml penicillin, and 100 mg/ml streptomycin at 37 °C and 5% CO_2_ 95% air environment. For experiments, cells were seeded at a density of 2×10^4^/ml in the 24-well plastic plates with a glass coverslips.

### Calcein loading of cells and iron influx assay

MES23.5 cells were divided into four groups: Control group, MPP^+^ group, MPP^+^ with isradipine treatment group, and MPP^+^ with Bayk8644 treatment group. Ferrous iron influx into MES23.5 cells was determined by the quenching of calcein fluorescence as described before [36, 37]. Control group: MES23.5 cells were cultured in serum-free DMEM/F12 medium for 24 h. MPP^+^ group: MES23.5 Cells were cultured in serum-free DMEM/F12 medium with 200 μmol/L MPP^+^ for 24 h. The cells were incubated with calcein-AM at a final concentration of 1 mmol/L in Hepes-buffered saline (HBS; 10 mmol/L Hepes, 150 mmol/L NaCl, pH 6.8) for 30 min at 37°C. Excess calcein on cell surface was washed out 3 times with HBS. The coverslips were mounted in a perfused chamber. 488 nm excitation and 525 nm emission wavelengths were used to record calcein fluorescence. Fluorescence intensity was measured every 3 min for 30 min while perfusing with 1 mmol/L ferrous iron (ferrous sulfate in ascorbic acid solution, 1:44 molar ratio, pH 6.0) in control and MPP^+^ groups. In the MPP^+^ with isradipine and MPP^+^ with Bayk8644 groups, MPP^+^ treated cells undergoing same procedures except that 0.02 mmol/L isradipine or 0.01 mmol/L Bayk8644 were included in the perfusing fluid. The mean fluorescence intensity of 25–30 single cell in four separate fields was monitored at 200 × magnification and processed with Fluoview 5.0 Software [38]. Data represent the means of three independent experiments.

### Statistical analysis

The results were represented as means ± SEM. The data were analyzed by two-way ANOVA followed by Bonferroni post hoc comparison of the means by using GraphPad 5.0 software (Graphpad Software, USA). *P* values that were less than 0.05 were considered to be significant.
